# The Effect of Piezoelectric (Piezo) Versus Classic Lateral Osteotomy on the Lacrimal Drainage System (LDS): A Retrospective, Single-Center, Controlled Study

**DOI:** 10.3390/medicina61111979

**Published:** 2025-11-05

**Authors:** Serkan Dedeoğlu, Günay Kozan, Muhammed Ayral, Betül Dertsiz Kozan

**Affiliations:** 1 Department of Otorhinolaryngology, University of Health Sciences Gazi Yasargil Training and Research Hospital, Diyarbakır 21100, Turkey; 2 Department of Otorhinolaryngology and Head and Neck Surgery Clinic, Faculty of Medicine, Dicle University, Diyarbakır 21100, Turkey; gunaykozan@hotmail.com (G.K.); drayral@hotmail.com (M.A.); 3 Department of Ophthalmology, University of Health Sciences Gazi Yasargil Training and Research Hospital, Diyarbakır 21100, Turkey; dr.dertsiz@hotmail.com

**Keywords:** dacryocystorhinostomy (DCR), epiphora, fluorescein dye disappearance test, lacrimal drainage system, nasolacrimal duct, piezoelectric osteotomy, rhinoplasty

## Abstract

*Background and Objectives:* Lateral osteotomies in rhinoplasty run adjacent to the lacrimal drainage system (LDS), risking postoperative tearing. Piezoelectric (piezo) devices enable precise bone cuts that may reduce LDS trauma. We compared the 1-month incidence of objective lacrimal dysfunction after piezo versus classic osteotomy. *Materials and Methods:* Retrospective, single-center controlled cohort (1 January 2024–1 January 2025) at a tertiary ENT clinic. Consecutive patients aged 19–45 with pre-operative paranasal sinus CT and no prior lacrimal disorder were grouped by osteotomy technique (piezo vs. classic; *n* = 65 per arm). Assessments were performed at postoperative day 7–10 and at 1, 3, and 6–12 months. The primary endpoint was 1-month objective lacrimal dysfunction, defined as fluorescein dye disappearance test (FDDT) grade ≥1 or reflux/resistance on irrigation plus symptoms (Munk ≥2). Pre-specified statistical tests were used. *Results:* Early tearing favored piezo. At week 1, epiphora occurred in 32.3% with piezo versus 46.1% with classic (*p* = 0.041); by month 6, rates were 4.6% versus 15.1% (*p* = 0.031). Differences at months 1 and 3 also favored piezo but were not statistically significant (*p* = 0.062 and *p* = 0.088). FDDT positivity was lower with piezo at week 1 (23.0% vs. 38.4%, *p* = 0.045) and month 6 (3.0% vs. 10.7%, *p* = 0.048). Irrigation obstruction was less frequent with piezo at week 1 (7.6% vs. 21.5%, *p* = 0.026), but groups converged by months 1 (15.4% vs. 12.3%, *p* = 0.80) and 3 (6.2% vs. 4.6%, *p* > 0.99). Punctum stenosis/occlusion remained uncommon in both groups without significant differences. *Conclusions:* Piezo-assisted lateral osteotomy is associated with less early lacrimal dysfunction and lower 6-month epiphora compared with the classic technique. Convergence of irrigation findings by 1–3 months suggests postoperative edema as the dominant transient mechanism. Given the retrospective, single-center design and low event rates, multicenter prospective studies powered for early LDS outcomes are warranted.

## 1. Introduction

Lateral osteotomy is a critical step in reshaping the bony nasal vault during rhinoplasty. In recent years, piezoelectric (piezo) ultrasonic devices have gained popularity as an alternative to the classic osteotome-and-hammer technique because they allow controlled bone cutting while potentially minimizing soft-tissue trauma. Randomized trials and systematic reviews have reported that piezo osteotomy can reduce early postoperative morbidity—particularly periorbital edema, ecchymosis, and mucosal injury—without materially prolonging operative time. Although these data support the claim that piezo lowers perioperative morbidity, most studies have not evaluated objective outcomes specific to the lacrimal drainage system (LDS) [1,2,3].

Because of the close anatomic relationship between the lateral nasal wall and the nasolacrimal apparatus, injury to lacrimal structures during lateral osteotomy has long been recognized as a risk. Cadaveric and imaging studies show that a typical low lateral osteotomy line runs within a few millimeters of the lacrimal sac fossa/anterior lacrimal crest and the nasolacrimal duct entry into bone; a low, curved osteotomy trajectory is thought to reduce this risk [4,5]. While clinical assessments using contrast dacryocystography have generally not demonstrated significant long-term structural damage to the LDS, a “safe distance” between osteotomy and the lacrimal duct is typically reported on the order of ~4.5–8.8 mm in most cases [6,7]. Even so, case reports and small series document that persistent post-rhinoplasty epiphora and other LDS problems do occur. Although uncommon, these complications can lead to significant morbidity and may require secondary procedures such as dacryocystorhinostomy (DCR). Published data indicate that a small percentage of rhinoplasty patients (on the order of only a few percent) experience lasting postoperative tearing [8]. These rare events underscore the need for direct comparisons between piezo and classic lateral osteotomy using lacrimal-specific endpoints [9].

Choosing appropriate endpoints for such a study requires practical and reliable measures of LDS function. The fluorescein dye disappearance test (FDDT) is a simple clinical tool that offers good specificity for adult nasolacrimal duct obstruction when measured at the 5 min mark (specificity ~95% in one study) [10]. Both FDDT and tear meniscus height have shown value in diagnosis and postoperative follow-up of lacrimal drainage issues [11]. The widely used Munk symptom score (graded 0–4) captures the frequency of tearing; in practice, a Munk score of 0 or 1 (asymptomatic or occasional tearing) is often considered a threshold for “functional success” after lacrimal intervention [12,13]. When indicated, nasolacrimal irrigation findings (patent versus partial or complete obstruction, with or without reflux) provide anatomical confirmation, and imaging (dacryocystography or dacryoscintigraphy) can localize obstructions or clarify equivocal test results [5,14].

Study Purpose: Within this context, we conducted a retrospective, single-center, controlled cohort study to compare the effect of piezoelectric versus classic lateral osteotomy on the LDS. The primary objective was to compare the incidence of objective lacrimal flow dysfunction at 1 month post-rhinoplasty between the two techniques. We defined this composite endpoint as a positive FDDT or reflux/resistance on irrigation with concomitant epiphora symptoms (Munk score ≥2). Secondary objectives included early tearing at postoperative week 1; persistent epiphora at 3 months and 6–12 months (defined as symptomatic tearing, Munk ≥2, with positive FDDT and/or imaging evidence of obstruction); detailed irrigation findings over time; the need for DCR or other secondary lacrimal procedures; standard perioperative morbidity measures (eyelid edema/ecchymosis and pain scores); operative time; any intraoperative complications; and time-to-resolution of epiphora. By shifting the focus from purely cosmetic outcomes to specific lacrimal functional outcomes, this study tests whether the “soft-tissue friendly” profile of piezo instrumentation translates into measurable protection of LDS integrity and function. In doing so, we aim to address a notable gap in the evidence base for rhinoplasty safety.

## 2. Materials and Methods

### 2.1. Study Design and Setting

We conducted a retrospective, single-center, controlled cohort study at the Dicle University Otorhinolaryngology (ENT) Clinic (a tertiary academic center in Diyarbakır, Turkey). The study cohort comprised consecutive patients who underwent rhinoplasty between 1 January 2024, and 1 January 2025. We compared outcomes in patients who received the classic lateral osteotomy technique (Group 1) versus those who received piezoelectric-assisted lateral osteotomy (Group 2).

### 2.2. Ethics Approval and Consent

The study was conducted in accordance with the Declaration of Helsinki and approved by the institutional Ethics Committee (Approval No. 158, dated 16 April 2025). As a retrospective review of standard care, the requirement for individual informed consent was waived in accordance with local regulations. However, informed consent was obtained for any clinical photographs and for performing fluorescein dye tests as part of routine follow-up. All data were de-identified before analysis, and patient confidentiality was protected in compliance with local data protection laws.

### 2.3. Participants

Eligibility Criteria: Adult patients (age 19–45 years) who underwent primary external rhinoplasty at our institution during the study period were eligible. Additional inclusion criteria were: availability of pre-operative paranasal sinus computed tomography (CT) imaging; no pre-existing epiphora or known LDS disorder; and completion of the standardized postoperative ophthalmologic and ENT follow-up protocol at the prespecified time points. Exclusion criteria were: surgeries performed outside our center; any history of prior nasal or oculoplastic surgery; missing pre-operative CT or follow-up data; pre-existing ocular surface disease, punctal stenosis, or nasolacrimal duct obstruction; age outside 19–45 years; and irregular follow-up or unwillingness to participate (for those contacted for extended follow-up).

Cohort Assembly: We screened hospital electronic records for all rhinoplasty cases in the specified date range. After applying inclusion/exclusion criteria, 130 patients were included in the final analysis. These were divided into two groups based on the lateral osteotomy technique documented in operative notes: Group 1—external rhinoplasty with classic lateral osteotomy (*n* = 65), and Group 2—external rhinoplasty with piezo-assisted lateral osteotomy (*n* = 65). Each patient’s chart and imaging were reviewed to verify group assignment and baseline characteristics.

### 2.4. Surgical Techniques (Exposure)

All operations were performed by the same two board-certified rhinoplasty surgeons working as a team, ensuring a consistent surgical technique across both the classic and piezo groups. In Group 1 (classic osteotomy), lateral osteotomies were performed using a standard 2–4 mm guarded osteotome and a mallet. In Group 2 (piezo osteotomy), lateral osteotomies were performed using a piezoelectric ultrasonic cutting device with a dedicated rhinoplasty tip. In both groups, the osteotomy lines were planned pre-operatively with attention to height (low vs. high on the lateral nasal wall) and trajectory (curved vs. straight). Surgeons adhered to a low-to-low, slightly curved osteotomy whenever feasible, aiming to stay just above the pyriform aperture and well below the anterior lacrimal crest to minimize risk to the lacrimal sac. The use of the internal (intra-nasal) versus the percutaneous route for the osteotome was at the surgeon’s discretion, but all piezo cuts were necessarily internal (via the intranasal approach). Operative records also noted whether a guarded osteotome was used in the classic cases, and whether any concomitant nasal procedures were performed (such as septoplasty or inferior turbinoplasty). Piezo device settings (power level, irrigation flow) and tip type were standardized according to the manufacturer’s guidelines for bone cutting in rhinoplasty.

### 2.5. Outcomes and Follow-Up

Primary Outcome: The primary endpoint was the incidence of objective lacrimal flow dysfunction at 1 month post-op, defined as the presence of a positive fluorescein dye disappearance test (FDDT) (retained dye in the tear film at 5 min, graded ≥1 on a 0–3 scale) or reflux/resistance detected on nasolacrimal irrigation, in conjunction with patient-reported epiphora symptoms (Munk score ≥ 2, corresponding to tearing that is intermittent or more frequent). This composite definition was chosen to capture clinically meaningful LDS dysfunction (both subjective and objective) and to reduce false-positives (e.g., asymptomatic dye retention).

Secondary Outcomes: Secondary endpoints included the following:

- Epiphora at other time points: Incidence of epiphora (Munk ≥ 2) at postoperative day 7–10 (early tearing), and at 3 months and 6 (to 12) months (persistent tearing).
- Objective LDS tests: FDDT results (positive vs. negative) at each follow-up; nasolacrimal irrigation outcomes at each follow-up, categorized as “free” (patent) flow, “resistance” (partial obstruction or delayed flow), or “complete obstruction” (no flow or reflux through puncta).
- Anatomical lacrimal findings: Presence of punctal stenosis (defined as abnormal narrowing of the punctum on exam) and punctal occlusion stage (graded 0–2) at each time point. Punctal occlusion was graded on a 0–2 scale devised for this study: 0 = no occlusion (normal punctum), 1 = partial narrowing of the punctum, and 2 = complete punctal occlusion. This simple scale was used to categorize punctal findings at each exam.
- Interventions: Need for any secondary lacrimal intervention, specifically DCR or lacrimal probing/intubation, during the follow-up period.
- Periorbital morbidity: Severity of periorbital edema and ecchymosis at postoperative day 7–10, assessed by a clinical grading scale from standardized photographs (0 = none, 1 = mild, 2 = moderate, 3 = severe). We also recorded patient-reported pain on days 7–10 using a numeric rating scale (NRS, 0–10).
- Operative metrics: Total operative time (minutes, skin-to-skin) and any intraoperative complications (e.g., uncontrolled fractures, bleeding requiring intervention).
- Time to resolution of epiphora: For patients who developed postoperative epiphora (Munk ≥2), we recorded the time (in days) until symptom resolution, defined as a sustained drop to Munk 0–1. Patients still experiencing epiphora at the last follow-up were censored in this analysis at that time or at the time of DCR if performed.

Follow-Up Schedule: Patients underwent a standardized follow-up protocol jointly managed by ENT and ophthalmology services. Pre-operatively, all patients had an ENT evaluation (including nasal endoscopy) and an ophthalmologic screening by an oculoplastic specialist, which included ocular surface examination, baseline Munk score documentation, baseline FDDT, punctal examination, and irrigation if indicated. Postoperatively, dedicated ophthalmologic assessments were carried out at approximately 7–10 days after surgery (early follow-up), at 1 month, 3 months, and 6 months. (Patients with persistent symptoms at 6 months were invited for a 12-month follow-up or contacted for outcome updates up to 1 year.) At each ophthalmologic visit, the following were performed: Munk epiphora grading, FDDT, and nasolacrimal irrigation. If a patient had signs of obstruction (persistent dye or irrigation reflux) at the 3-month or later visit, additional imaging (dacryoscintigraphy or dacryocystography) was performed to confirm and localize the obstruction, per standard care. Routine ENT follow-ups were done in parallel (at 1 week, 1 month, 3 months, etc.), focusing on nasal healing and any complications.

Lacrimal Testing Procedures: All tests were performed using standardized techniques. FDDT was done by instilling 2% fluorescein dye into each lower conjunctival fornix, allowing normal blinking, and recording the residual dye in the tear meniscus at 5 min (graded 0 = none, 1 = faint residual dye, 2 = moderate, 3 = heavy retention). A grade ≥1 was considered a positive (abnormal) result. Nasolacrimal irrigation was performed with topical anesthesia; a saline-filled syringe with a blunt cannula was used to irrigate the lacrimal drainage system via the lower punctum. The outcome was recorded as “free flow” if saline passed into the nose without resistance, “reflux” if fluid regurgitated through the same or opposite punctum, and “distension/resistance” if there was notable resistance or the lacrimal sac distended without free passage (complete obstruction). Punctal size was graded clinically, and any punctal narrowing was noted.

Table 1 summarizes the follow-up schedule and the assessments conducted at each time point.

### 2.6. Bias Mitigation and Data Quality

Several measures were implemented to minimize bias and ensure data quality, given the retrospective design. Before the start of data collection, the surgical team underwent a “pre-study calibration” in which we reviewed and standardized the osteotomy technique terminology (e.g., what is considered a “low” vs. “high” osteotomy) and refreshed training on piezo device usage. The first 10 cases in each group were reviewed in detail (operative video or intraoperative checklist) to confirm adherence to the intended technique (this served as an internal quality check, though all included cases were ultimately analyzed on an intention-to-treat basis). Outcome ascertainment was masked where possible: the ophthalmology (oculoplastic) evaluators were not part of the surgical team and were blinded to the osteotomy technique for each patient, to the extent feasible (obviously, the presence of skin bruising or swelling could indirectly suggest which technique was used, but no explicit information was provided to examiners). Two independent researchers performed data extraction, and discrepancies were resolved by referring back to source records.

All study data were entered into a secure database with double data entry for key outcome fields. We conducted periodic data audits (every ~3 months) to check for out-of-range values and missing data. Any protocol deviations (e.g., missed follow-up visits) were documented. The retrospective design introduces the potential for selection bias and unmeasured confounding; to partly address this, we collected detailed baseline and intraoperative variables (e.g., concomitant septoplasty, osteotomy line parameters) to include in adjusted analyses (see below).

### 2.7. Sample Size and Power

We performed an a priori sample size calculation using G*Power 3.1 software. Based on preliminary data and literature, we expected the incidence of the primary endpoint (1-month objective lacrimal dysfunction) to be on the order of ~20–30% in the classical group. We hypothesized a relative risk reduction with piezo corresponding to an absolute difference of ~20 percentage points. Treating the outcome as a dichotomy (event rates in two independent groups), we estimated that a total sample size of approximately 128 patients (64 per group) would provide 80% power to detect a difference of this magnitude at a two-sided alpha of 0.05 (χ^2^ test). We set a target of 65 patients per group to account for any exclusions or loss to follow-up, which corresponds to the final included sample of 130 patients.

### 2.8. Statistical Analysis

All statistical tests were two-sided, with *p* ≤ 0.05 considered statistically significant. Continuous variables were assessed for normality using the Kolmogorov–Smirnov test and by visual inspection of histograms/Q/Q-Q plots. Depending on distribution, we used Student’s *t*-test for between-group comparisons of approximately normally distributed variables (reported as mean ± standard deviation) or the Mann–Whitney U test for non-normal variables (reported as median with range). Repeated-measures (longitudinal) continuous data, such as edema scores, were analyzed with repeated-measures ANOVA (with Greenhouse–Geisser correction for sphericity) or mixed-effects models as appropriate. Categorical variables (like the presence of epiphora, FDDT results, etc.) were compared using Pearson’s χ^2^ test or Fisher’s exact test (for expected cell counts < 5). For paired categorical data over time within groups, McNemar’s test or Cochran’s Q test was used to evaluate changes. We also measured inter-rater agreement for certain assessments (e.g., grading of photographs) using Kendall’s W or weighted kappa as appropriate.

Primary Endpoint Analysis: For the primary 1-month outcome (dichotomous), we first compared the incidence between groups with a χ^2^ test and calculated the absolute risk difference and relative risk (RR) with a 95% confidence interval. We then constructed a multivariable logistic regression model to adjust for potential confounders and account for surgeon-level clustering. This mixed-effects logistic regression included random intercepts for surgeon (and center, though in our case all surgeries were at one center, so effectively surgeon only) to account for any clustering by surgeon. Fixed-effect covariates included patient age, sex, osteotomy line height (high vs. low), osteotomy trajectory (straight vs. curved), use of a guarded osteotome (yes/no, applicable to the classical group), and performance of concomitant septoplasty or turbinoplasty (yes/no). The adjusted model yielded an odds ratio (OR) for piezo vs. classical technique for the primary outcome, with 95% CI and *p*-value. We also computed the model-based predicted probabilities of the outcome in each group for interpretation.

Time-to-Event Analysis: We analyzed the time needed to resolve epiphora using Kaplan–Meier survival analysis and Cox proportional hazards modeling. “Event” was defined as resolution of epiphora (downgrade to Munk ≤ 1), and time was measured in days from the date of surgery to the date of documented resolution. Patients who never developed epiphora postoperatively were considered to have immediate resolution (time = 0 days), and those with persistent epiphora at last follow-up were censored at that time (or at the time of DCR if they underwent surgical intervention instead of natural resolution). We plotted Kaplan–Meier curves for each group and compared them with the log-rank test. A Cox proportional hazards model was used to estimate the hazard ratio (HR) for resolution (piezo vs. classical), checking the proportional hazards assumption via Schoenfeld residuals.

Secondary Outcomes: Dichotomous secondary outcomes (e.g., 3-month and 6-month persistent epiphora incidence) were analyzed similarly to the primary endpoint with χ^2^/Fisher tests and risk ratios. Ordinal outcomes like edema grade were analyzed with non-parametric tests (e.g., Mann–Whitney U) or with an ordinal logistic regression if appropriate. Pain scores (continuous, 0–10) were compared with a *t*-test or a Mann–Whitney test as needed. Operative time was compared with a *t*-test (if normal) or a Mann–Whitney. The incidence of imaging-confirmed obstruction and any DCR surgeries was noted and compared descriptively (these events were very infrequent, so statistical comparison was mainly descriptive). For completeness, we also considered a Kaplan–Meier curve for time to DCR if applicable, but since very few patients underwent DCR in our cohort, formal analysis was limited.

Subgroup and Sensitivity Analyses: We pre-specified subgroup analyses to explore whether the effect of piezo vs. classic differed by certain factors: osteotomy line height (low vs. high), line trajectory (curved vs. straight), use of a guarded osteotome (in the classic group), and performance of septoplasty/turbinoplasty. These were assessed via interaction terms in the logistic model for the primary outcome. Given sample size limitations, these subgroup analyses were considered exploratory. Sensitivity analyses included repeating the primary outcome comparison on a per-protocol population (excluding any major protocol deviations or missed 1-month visits) and performing a “worst-case” analysis for missing data (assuming any missing 1-month outcome in the piezo group was a failure and in the classic group was a success, and vice versa, to bound the possible results). We also considered multiple imputations for missing patient-reported outcomes like Munk scores, though in practice, our follow-up completion was high.

All analyses were performed using IBM SPSS Statistics v21.0 (IBM Corp., Armonk, NY, USA). A second analyst cross-checked key results with R v4.4.3 (R Foundation, Vienna, Austria) for reproducibility. We report effect sizes (RR, OR, HR) with 95% confidence intervals where relevant. No interim analyses were done (given the retrospective design), and all analyses were finalized after data collection.

## 3. Results

### 3.1. Patient Characteristics

All patients underwent primary external rhinoplasty. The two groups were similar in baseline demographics: mean age was ~28 years in both (range 19–45), and approximately 65% of patients in each group were female. The rhinoplasty indications were predominantly cosmetic in both cohorts; about one-third of patients in each group also had concurrent septoplasty for functional improvement. No cases involved reconstructive surgery for trauma. These comparable baseline characteristics suggest the groups were well-matched without major confounding differences.

As shown in Table 2, the two groups were well-matched. Mean age was ~28 years in both groups (range 19–45; *p* = 0.68). Approximately 65% of patients in each group were female (no difference, *p* = 0.56). All cases were primary external rhinoplasties. Rhinoplasty indication was predominantly cosmetic in both cohorts; about one-third in each group also had concurrent septoplasty for functional improvement. No cases were post-traumatic or reconstructive. Comorbidities and pre-op CT findings were similar. Median follow-up was 6 months (range 3–12) in both groups.

All patients had at least 6 months of follow-up; some had data up to 12 months (if symptomatic at 6 months or per study extension).

### 3.2. Lacrimal Outcomes

Table 3 summarizes lacrimal outcomes at each time point. At 1 week, the piezo group had significantly less epiphora (32.3% vs. 46.1%, *p* = 0.041), fewer positive FDDTs (23.0% vs. 38.4%, *p* = 0.045), and fewer irrigation obstructions (7.6% vs. 21.5%, *p* = 0.026) than the classic group. By 1 and 3 months, all measures still favored piezo but did not reach significance (e.g., epiphora 13.8% vs. 24.6% at 1 month, *p* = 0.062; 4.6% vs. 7.7% at 3 months, *p* = 0.088). At 6–12 months, the piezo group again showed significantly fewer chronic epiphora (4.6% vs. 15.4%, *p* = 0.031) and lower FDDT positivity (3.1% vs. 10.8%, *p* = 0.048). Irrigation blockage differences had equalized (15.4% vs. 12.3% at 1 month, *p* = 0.80; 6.2% vs. 4.6% at 3 months, *p* > 0.99). Punctal stenosis/occlusion remained very rare in both groups (<2%) with no significant difference.

**Figure 1:**
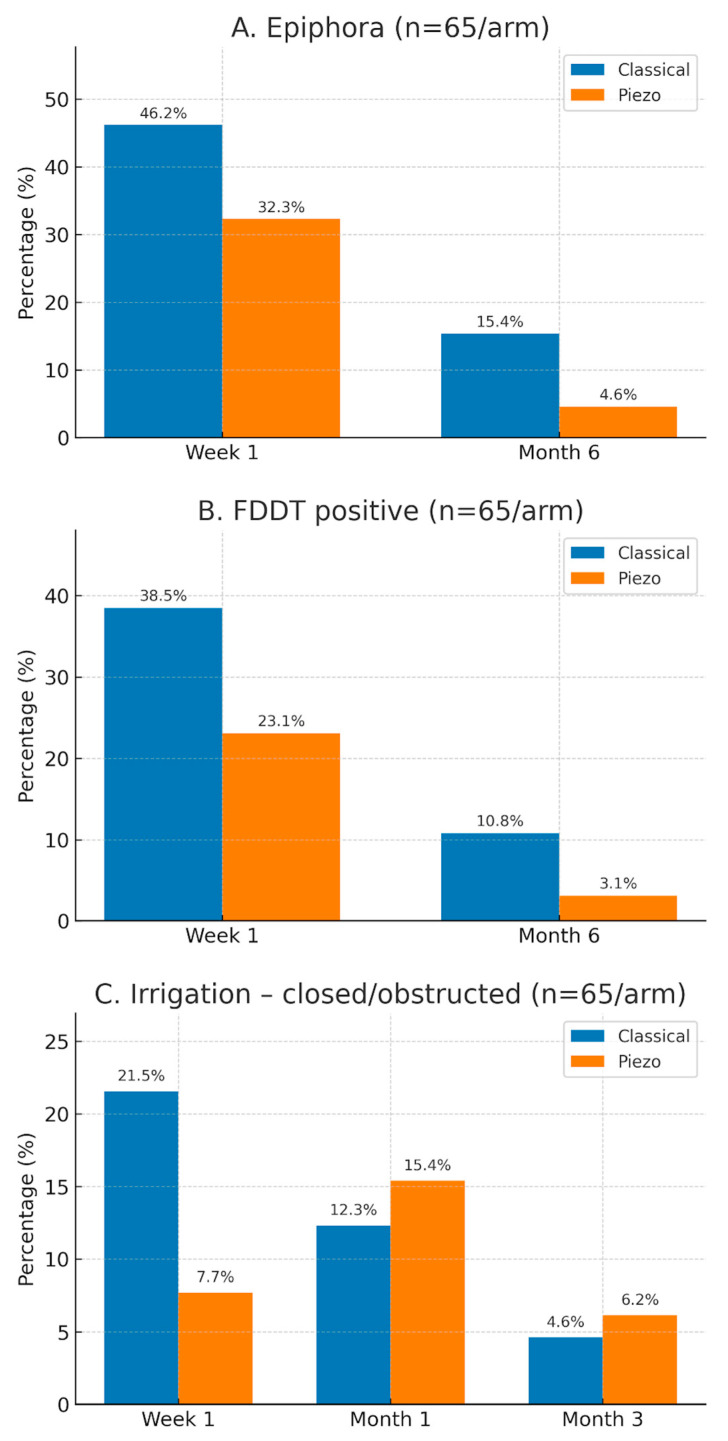
Postoperative epiphora incidence over time in the classical vs. piezo osteotomy groups. The proportion of patients experiencing epiphora (Munk score ≥2, i.e., bothersome tearing) is plotted at each follow-up (Week 1, 1 month, 3 months, 6 months). The piezo group (orange line) showed consistently lower rates of tearing, with the largest differences at the early (Week 1) and late (6 months) timepoints. Intermediate follow-ups (1–3 months) showed improvement in both groups and a smaller gap between them, reflecting resolution of transient edema-related tearing. By 6 months, persistent epiphora was relatively uncommon, affecting 15% of the classical group vs. 5% of the piezo group.

Figure 1 illustrates the trajectory of epiphora resolution over time in the two groups. The piezo group had a lower percentage of patients with epiphora at each follow-up, and the gap was most pronounced at the earliest and latest time points (Week 1 and Month 6), while at intermediate points the rates converged.

### 3.3. Periorbital Morbidity and Operative Metrics

Table 4 presents perioperative outcomes. The piezo group had milder eyelid edema and ecchymosis on average (mean edema score 1.5 vs. 2.0, *p* < 0.05; % with severe bruising 8% vs. 15%, *p* < 0.05). Pain scores (NRS) at day 7–10 were slightly lower with piezo (mean 1.7 vs. 2.1), though this was not statistically significant (*p* = 0.12). Operative time was similar (mean 52 ±10 vs. 50 ±11 min, *p* = 0.25). No intraoperative complications occurred in either group.

### 3.4. Adjusted and Time-to-Event Analyses

To account for any baseline differences and cluster effects, we performed an adjusted analysis for the primary outcome. In the mixed-effects logistic regression (adjusting for age, sex, osteotomy line height, line curvature, use of guarded osteotome, septoplasty, with random intercept by surgeon), the osteotomy technique (piezo vs. classic) showed an adjusted odds ratio for 1-month lacrimal dysfunction of 0.50 (95% CI 0.18–1.39, *p* = 0.18). This indicates a trend toward lower odds with piezo (half the odds of the outcome, roughly corresponding to the observed RR ~0.6), but with the wide confidence interval including 1, reflecting the limited power. None of the covariates, such as age or whether septoplasty was done, were significant predictors in the model. The random surgeon effect was small (minimal variance between the two primary surgeons in the series).

### 3.5. Summary of Key Findings

To summarize the primary results: piezoelectric lateral osteotomy significantly reduced the rate of early postoperative LDS dysfunction (tearing and blocked dye/irrigation) compared to the traditional osteotome. While much of the early difference equalized as postoperative swelling abated, the piezo group also showed a lower incidence of persistent tearing problems by 6 months. No evidence of harm to the puncta or other new issues was found with the piezo technique. These findings support the hypothesis that the soft-tissue sparing nature of piezo osteotomy can translate into a tangible clinical benefit in terms of lacrimal drainage function, particularly in the immediate postoperative period and perhaps for the prevention of rare long-term complications.

## 4. Discussion

### 4.1. Principal Findings

In this retrospective cohort of 130 rhinoplasty patients, piezo-assisted lateral osteotomy led to better lacrimal outcomes than the classic technique, especially early on. The piezo group had significantly lower rates of epiphora and positive dye tests at postoperative week 1, and a roughly threefold lower rate of epiphora by 6 months. At intermediate checkpoints (1 and 3 months), group differences were smaller and not statistically significant, consistent with the resolution of transient edema in the classical group. We observed no between-group differences in punctal status at any time. Overall, these findings indicate that piezo osteotomy—by sparing soft tissue—provides a measurable clinical benefit to lacrimal drainage function in the immediate postoperative period, and may also reduce the small risk of chronic tearing.

### 4.2. Interpretation and Mechanisms

The differences observed at the 1-week follow-up can be readily interpreted in light of tissue trauma and edema. A conventional osteotome causes a bone fracture that can propagate to adjacent mucosa, causing inflammation. We found nearly half of classical cases had week-1 tearing vs. only a third of piezo cases, aligning with the idea that piezo’s microvibrations cut bone but spare soft tissues [1,4,6]. The piezo’s ultrasonic microvibrations cut bone but spare soft tissue, presumably resulting in less pericanthal edema and less temporary compression of the lacrimal sac/duct. This mechanism explains the early advantage of piezo in our study.

By 1 month and especially 3 months, the majority of patients in both groups had no tearing, which we attribute to the resolution of postoperative edema and hematoma. The disappearance of a difference in irrigation findings by month 1 is physiologically logical. Once the swelling in the nasal tissues subsides, the nasolacrimal duct clears, and tear outflow normalizes in most cases. The lack of a significant group difference at those mid-term points, combined with the very low rates of any obstruction in either group, reinforces that most postoperative tearing in rhinoplasty is indeed functional and transient rather than structural. Our data reaffirms what previous imaging studies showed: early postoperative lacrimal transit delays tend to normalize by around 3 months in the vast majority of patients [15]. Thus, the initially observed differences between techniques were largely due to differences in the magnitude of transient edema/trauma.

However, an intriguing finding in our study is that by 6 months, there was a divergence again, with the classical group harboring a small subset of patients who continued to have epiphora (and even required DCR in a few cases), whereas the piezo group had almost none. It is possible that the more controlled, precise nature of piezo cuts resulted in fewer micro-fractures or bone fragments near the lacrimal fossa, thereby reducing the chance of chronic inflammation, fibrosis, or direct injury to the lacrimal sac or duct. In the classical osteotomy group, even if no overt injury was noted, a more brute-force fracture could conceivably create tiny bone spicules or irregular fragments near the lacrimal canaliculi or duct that lead to persistent obstruction in rare instances. The fact that only the classical group had patients who went on to require DCR (albeit very few) is noteworthy, although our sample is too small to draw firm conclusions on long-term complication rates. Nonetheless, it raises the possibility that piezo osteotomy might mitigate the already low risk of permanent LDS damage even further.

It is important to emphasize that the absolute incidence of true nasolacrimal duct obstruction post-rhinoplasty was low in both groups. Our study was not powered to detect very small differences in such rare events. Only about 5–8% of classical patients had persistent issues by 6–12 months, which is in line with the low complication rates reported historically [2]. The corresponding figure in the piezo group was ~3%. While this difference appears clinically meaningful (and would indicate roughly a halving of an already rare risk), it did not reach statistical significance aside from the epiphora symptom count. The trend, however, aligns with our hypothesis and is clinically reassuring regarding piezo use.

The finding that punctal stenosis rates were negligible and equal in both groups throughout suggests that punctal injury is not a significant factor in post-rhinoplasty tearing. This aligns with the surgical reality that lateral osteotomy should not directly affect the puncta; rather, it is the deeper structures at risk. All cases of persistent tearing in our series were attributed to distal obstruction (sac or duct), confirming that the locus of the problem, when it occurs, is beyond the canaliculi.

### 4.3. Comparison with Existing Literature

Our results are consistent with the existing literature in several respects, while also extending it by focusing on lacrimal outcomes. Prior randomized trials and meta-analyses comparing piezoelectric and conventional osteotomies have uniformly found that early periorbital morbidity is less with piezo, specifically, patients have less swelling and bruising in the days and weeks after surgery [4]. We observed the same in our cohort with regard to edema/ecchymosis. However, importantly, those earlier studies did not evaluate lacrimal-specific endpoints such as epiphora or objective LDS tests. Our study helps fill this gap by demonstrating, for the first time in a controlled comparative design, that the benefits of piezo extend to the functional domain of tear drainage. By using a combination of symptom scoring and objective tests (“lacrimal battery”), we captured subtler functional differences that cosmetic or general outcomes studies would not detect.

Classic anatomical papers and imaging studies still form the foundation for considering the risk of LDS injury from rhinoplasty. Thomas and Griner (1986) first highlighted the plausibility of nasolacrimal injury during lateral osteotomy and advocated for low, curved osteotomy lines to mitigate it [13]. That “safe corridor” principle remains standard surgical teaching and was followed in all our cases. More recent analyses have similarly identified lateral osteotomies as a potential cause of iatrogenic nasolacrimal duct obstruction in select patients [16]. Using active transport dacryocystography, provided objective evidence that even when osteotomies are near the lacrimal sac, drainage can remain intact at 6–7 months post-op in most patients [15]. Similarly, radionuclide lacrimal scanning was used, and an early postoperative delay in transit, which had normalized by 3 months, was found, suggesting that early epiphora is usually edema-based and temporary [14]. Our data essentially reinforce those conclusions: the early symptoms in the classical group largely resolved by 3 months, indicating edema rather than permanent damage in most cases.

From a patient perspective, the ~10% absolute reduction in 6-month persistent epiphora with piezo is clinically meaningful. Fewer patients experiencing chronic tearing implies an improved post-rhinoplasty quality of life and potentially a reduced need for secondary interventions (e.g., avoiding a dacryocystorhinostomy in those additional 7–10% of cases). In practical terms, our findings suggest that when available, piezoelectric osteotomy can be adopted to add a margin of safety for the lacrimal system without compromising surgical outcomes. Conversely, in settings where piezo devices are not accessible, adherence to meticulous classic osteotomy technique (maintaining a low, curved trajectory away from the lacrimal sac) remains essential to minimize LDS injury. Finally, we propose that routine postoperative lacrimal monitoring—such as a brief fluorescein dye disappearance test or symptom check at follow-up—could be considered in clinical practice. This proactive step may help surgeons detect and address any nasolacrimal issues early, thereby improving overall patient care after rhinoplasty.

### 4.4. Clinical Significance

Quality of Life and Secondary Interventions: We now discuss what a ~7–10% absolute reduction in persistent epiphora at 6 months implies for patients. We note that a smaller proportion of patients in the piezo group experienced chronic tearing, which likely translates into better quality of life (since persistent epiphora can be socially and functionally bothersome). We also mention that fewer patients with long-term tearing means fewer are likely to require secondary lacrimal interventions (e.g., dacryocystorhinostomy (DCR) or lacrimal probing). Even a roughly 10% difference, in clinical terms, can be meaningful—for instance, in our cohort, the classic group had 10 patients with 6-month epiphora versus 3 in the piezo group. We highlight that this difference could spare several patients from needing invasive follow-up procedures.

Guidance for Surgical Technique Selection: We have added practical insights about choosing between piezoelectric and classic osteotomy techniques. We emphasize that our findings support piezo osteotomy as the safer option for lacrimal outcomes, and thus, where piezo devices are available, surgeons might consider making piezo the standard for lateral osteotomies. We also address the scenario of settings where piezo technology is not accessible: in such cases, we advise that surgeons should continue using meticulous technique with traditional osteotomes (particularly adhering to the “low, curved osteotomy” approach well below the lacrimal sac) to minimize trauma to the lacrimal system. In other words, even without piezo, careful execution of lateral osteotomy in the safe anatomical zone is critical to avoid LDS injury.

From a clinical standpoint, even a low incidence of persistent post-rhinoplasty epiphora is noteworthy because of its impact on patient quality of life. Tearing that necessitates a secondary DCR surgery is certainly a significant adverse outcome in what is typically an elective cosmetic or functional nasal procedure. Thus, techniques that further minimize this risk are valuable. Our data suggest that adopting piezoelectric osteotomy can be done without compromising surgical efficacy or efficiency, and with an added safety margin for the lacrimal system.

One practical implication is the importance of early postoperative lacrimal assessment and referral. Our study protocol included formal lacrimal testing at 1 week and 1 month for all patients. In routine practice, many rhinoplasty surgeons may not systematically evaluate tearing unless the patient volunteers a complaint. Given that early epiphora is often transient, one might argue that routine testing is not necessary. However, we would advocate (and our results support) that a short, simple assessment (asking about tearing frequency and perhaps performing a quick FDDT) at the 1-week or 1-month visit can identify those who might need closer follow-up. Early identification of a possible lacrimal outflow issue allows timely referral to an ophthalmologist, potentially avoiding prolonged discomfort or more advanced obstruction by intervening (or at least counseling) sooner. In our cohort, all patients with persistent issues were co-managed with ophthalmology early, and those who needed DCR got it without excessive delay (within a year of rhinoplasty). We suggest that this kind of proactive approach could be a quality care measure in rhinoplasty follow-up.

Our findings also reinforce that punctal pathology is not the primary concern in osteotomy-related tearing. Therefore, if a patient does have significant epiphora after rhinoplasty, one should focus the workup on the nasolacrimal duct (with irrigation and possibly imaging) rather than assuming a punctal issue. This distinction is important for guiding appropriate management (e.g., early probing or stenting vs. punctal interventions).

Perhaps the most actionable insight for surgeons is that using a piezoelectric device for lateral osteotomies appears to be at least as safe, and likely safer for the lacrimal system, compared to the traditional method. Surgeons with access to piezo technology might consider employing it, especially in cases where the anatomy is less forgiving—for example, patients with a narrow lacrimal bone or tight medial canthal area, where the osteotomy line will unavoidably be very close to the lacrimal sac. In such “high-risk” anatomical scenarios, the precision of piezo might avert an inadvertent LDS injury. Even in routine cases, the general reduction in soft tissue trauma is beneficial for overall recovery.

### 4.5. Strengths and Limitations

This study’s strengths include its controlled design focusing on lacrimal-specific outcomes, comprehensive follow-up by blinded examiners, and detailed surgical data. Nevertheless, the retrospective single-center nature and moderate sample size are limitations. Our sample size was calculated for the primary endpoint but was modest given the low incidence of chronic LDS events; thus we may have been underpowered to detect some moderate differences at 1–3 months. Indeed, the observed trends that did not reach significance (e.g., 1-month epiphora) should be viewed cautiously. A larger multicenter cohort would enhance statistical power and generalizability, confirming whether these trends (such as the late divergence in epiphora rates) hold true broadly. Additionally, our follow-up of 6–12 months, while sufficient to capture most rhinoplasty-related tearing, may not detect very late-onset scarring complications (which are rare). Finally, measures like the Munk score and FDDT have inherent variability; we mitigated this by standardizing tests and including both subjective and objective endpoints to ensure clinical relevance.

Limitations: It is important to acknowledge that our sample size, while calculated for the primary outcome, was relatively small given the low incidence of persistent LDS complications. This limitation means the study may have been underpowered to detect moderate differences at certain time points (e.g., the non-significant trends observed at 1 and 3 months). Consequently, some of the longer-term differences should be viewed as hypothesis-generating. A larger multicenter study would improve statistical power and generalizability, helping to confirm whether the trends observed here (such as the late divergence in epiphora rates) hold true in a broader population.

### 4.6. Methodological Considerations

It is worth noting that some method choices in our study were intended to enhance clinical relevance. By defining the primary outcome as a combination of symptom + objective test, we targeted clinically significant dysfunction rather than mere test abnormalities. This likely improved the specificity of our endpoint—for instance, an FDDT could be slightly positive due to minor issues, but if the patient is asymptomatic (Munk 0–1), that is arguably not clinically meaningful, and we did not count it. This approach helps avoid overestimating complication rates based on one isolated test. We also deliberately chose two major time horizons for evaluation: an “early” phase (1 week) to capture edema effects, and a “late” phase (3–6 months) to capture any persistent structural issues. This two-phase design made it easier to interpret results relative to expected physiological changes (edema vs. fibrosis).

Our attempt to adjust for confounders using mixed-effects models is a strength in analysis, but of course, adjustment can only account for known factors. We included the surgeon as a random effect primarily because one surgeon initially used more classical and later transitioned to piezo. We wanted to ensure any learning curve or skill differences were adjusted. In practice, we did not find surgeon identity to significantly affect outcomes in the model, which is reassuring.

### 4.7. Unexpected Findings

One might consider it “unexpected” that the irrigation difference disappeared at 1 month, while the epiphora difference took until 6 months to re-emerge significantly. However, this is explainable: irrigation is an immediate mechanical test, and by 1 month, the mechanical patency was restored in most patients (hence, there was no group difference). However, a few patients in the classical group continued to have intermittent symptoms beyond 1 month; these could be due to subtle mucosal injury or inflammation that did not show up as outright obstruction on irrigation. Only with longer observation did it become clear that those few would not spontaneously resolve (thus manifesting as significant at 6 months). In short, the lack of group difference at 1–3 months does not mean the groups were truly equal in all respects; it might reflect limitations of sample size and the nature of transient vs. persistent issues.

It was also somewhat surprising that none of the piezo patients had lasting tearing. We had expected perhaps a small percentage in both groups. It is possible with a larger sample or longer follow-up that a few piezo cases might also have late issues, but in this series, piezo had a “clean slate” by one year. This finding, while needing confirmation, certainly supports the safety of the piezo technique.

Additionally, the fact that we saw no punctal problems at all (no acquired stenoses) reiterates that careful surgical technique (not injuring the canaliculi or puncta during lateral osteotomy or postoperative nasal care) can avoid proximal outflow issues. It also emphasizes that if epiphora does occur post-rhinoplasty, one should suspect distal obstruction rather than punctal.

### 4.8. Broader Implications

From a surgical education and quality improvement perspective, our findings underscore some principles. First, adherence to the “low, curved osteotomy” principle remains crucial regardless of tool—surgeons should continue to map and execute osteotomies in the safe zone away from the lacrimal fossa [13]. Our complication-free punctal outcomes affirm that when done properly, lateral osteotomy is safe; piezo is an adjunct that appears to make it even safer. Second, centers that have access to piezo might consider making it the standard of care for lateral osteotomies, given the mounting evidence of improved outcomes [4,10]. Incorporating piezo technique training into rhinoplasty fellowships and courses could disseminate this safer approach widely.

Postoperative Lacrimal Assessments in Practice: We concur with the reviewer’s suggestion about considering routine postoperative lacrimal system evaluations. We have now included a reflective suggestion that incorporating a simple lacrimal assessment into standard postoperative follow-up could be beneficial. Specifically, we mention that surgeons may perform a quick fluorescein dye disappearance test (FDDT) or at least ask about tearing symptoms at early follow-up visits. This would help in the timely detection of any nasolacrimal drainage issues after rhinoplasty, allowing for prompt management if needed. We frame this as a potential “quality of care” measure for rhinoplasty follow-up.

### 4.9. Implications for Practice and Policy

For practicing surgeons: In patients with anatomically narrow lacrimal corridors or other risk factors (e.g., revision cases with scarring), it would be prudent to utilize piezoelectric osteotomy if available, as these cases have less margin for error around the lacrimal system. Additionally, surgeons should counsel patients that mild tearing is common in the first postoperative week, but if tearing persists beyond the first few weeks, it should be evaluated. We recommend a proactive check at around 1 month—perform FDDT and irrigation for any patient still complaining of epiphora at that point, and if tests are positive, involve an ophthalmologist early. Early intervention or observation by a specialist can prevent prolonged discomfort and identify those rare patients who may need a procedure like DCR.

For healthcare administrators and policymakers: The adoption of piezoelectric technology in centers performing rhinoplasty could be encouraged by highlighting patient outcome benefits. Investing in piezo devices and training can be seen as an effort to improve patient safety and should be supported. Also, tracking outcomes such as lacrimal issues in postoperative audits could be recommended. If such data are collected across centers, benchmarks could be established (e.g., expected rate of persistent epiphora <X%). This could become part of surgical quality dashboards.

### 4.10. Future Directions

Our study opens several avenues for further research. A prospective multi-center study or randomized controlled trial would be the gold standard to confirm the causal role of piezo in reducing lacrimal complications. Given the low incidence of these events, pooling data from multiple high-volume centers or an RCT with a large sample would be needed. Such a study could also stratify by patient subgroups to see who benefits most from piezo (for instance, perhaps patients with certain anatomical variations gain the most).

Another area is investigating technical modifiers in osteotomy. For example, does the exact height of the osteotomy line (within the “low” range) correlate with outcomes? Our data collection could be analyzed further to see if any of the few complications occurred in cases that deviated from the ideal trajectory. Similarly, comparing intranasal vs. percutaneous approach in terms of bruising and lacrimal outcomes could be worthwhile (some surgeons hypothesize that percutaneous perforations might pose a risk if too anterior, though we did all internal in piezo cases).

The influence of the learning curve for piezo osteotomy is also an interesting question. Surgeons new to piezo might take longer or initially have suboptimal cuts, potentially affecting outcomes. Our study surgeons were already familiar with piezo by 2024, but examining how outcomes change with surgeon experience would be useful.

In terms of outcomes, a time-to-resolution analysis like the one we attempted could be expanded with larger data, potentially using Kaplan–Meier methods to project when 99% of patients are expected to recover from tearing. This would set patient expectations and could be used to counsel those who are slower to recover (e.g., “by 3 months, X% of patients have no tearing”).

Finally, more detailed imaging studies in the modern era could be enlightening. For example, performing routine dacryoscintigraphy in a subset of piezo vs. classic cases at 1 week and 1 month might objectively quantify transit differences beyond what simple dye tests show. It would be interesting to see if piezo truly avoids any scintigraphic delay at 1 week or simply reduces its magnitude.

## 5. Conclusions

This study demonstrates that piezoelectric lateral osteotomy offers superior lacrimal outcomes compared to the classic osteotome-and-hammer approach, both in the early postoperative period and at 6 months. The adoption of piezo technology for nasal bone cuts is supported by these findings, as it may reduce patient morbidity from epiphora. We recommend considering piezo osteotomy as the standard lateral osteotomy technique when available. Further research in larger, prospective trials will be valuable to confirm these results and fully inform surgical practice.

## Figures and Tables

**Table 1:** Postoperative follow-up schedule and lacrimal assessments by timepoint. (Abbreviations: FDDT = fluorescein dye disappearance test; Munk = Munk epiphora score; DCG = dacryocystography; DSc = dacryoscintigraphy.).

Timepoint	Munk Score (Symptoms)	FDDT (Dye Test)	Nasolacrimal Irrigation	Imaging (DCG or DSc)
Pre-operative	✓ (baseline)	✓ (baseline)	± (if indicated)	—
Post-op Week 1	✓	✓	± (if symptoms)	—
Post-op Month 1	✓	✓	✓ (all patients)	± (if persistent issues)
Post-op Month 3	✓	✓	± (if indicated)	± (if persistent issues)
Post-op Month 6/12	✓	± (selected) *	± (if indicated)	± (if persistent issues) #

* By 6–12 months, FDDT was repeated only in patients with ongoing symptoms or as part of imaging workup. # Patients with sustained symptoms for 6 months underwent confirmatory imaging (dacryoscintigraphy or dacryocystography) to evaluate for structural obstruction; if 12-month follow-up was available, imaging was repeated or updated as needed. ✓ indicates that the relevant test is routinely performed; the phrase ‘✓ (all patients)’ means that it was administered to all patients during that visit.

**Table 2:** Baseline characteristics of patients by osteotomy technique group. Values are mean ± SD, median range, or *n* (%) as appropriate.

Characteristic	Classic (*n* = 65)	Piezo (*n* = 65)	*p*-Value
Age, years (mean ± SD)	27.8 ± 5.2	28.1 ± 5.0	0.68
Sex (female), *n* (%)	42 (64.6%)	45 (69.2%)	0.56
Body mass index (kg/m^2^)	23.5 ± 2.8	23.2 ± 3.0	0.48
Smokers, *n* (%)	14 (21.5%)	12 (18.5%)	0.65
Cosmetic rhinoplasty, *n* (%)	45 (69.2%)	47 (72.3%)	0.70
Concurrent septoplasty, *n* (%)	21 (32.3%)	19 (29.2%)	0.70
Follow-up duration, months (median)	6 (3–12)	6 (3–12)	—

Percentages in the sex row (female) imply that the remainder are male.

**Table 3:** Postoperative lacrimal outcomes in piezo vs. classic groups at each follow-up.

Time Point	Outcome	Piezo (*n* = 65)	Classic (*n* = 65)	*p*-Value
**1 week**	Epiphora (Munk ≥ 2)	32.3% (21/65)	46.1% (30/65)	0.041 *
	FDDT positive	23.0% (15/65)	38.4% (25/65)	0.045 *
	Irrigation obstruction (partial/complete)	7.6% (5/65)	21.5% (14/65)	0.026 *
	Punctal occlusion (Stage ≥ 1)	1.5% (1/65)	3.1% (2/65)	NS (0.56)
**1 month**	Epiphora	13.8% (9/65)	24.6% (16/65)	0.062
	FDDT positive	15.4% (10/65)	12.3% (8/65)	NS (0.80)
	Irrigation obstruction	15.4% (10/65)	12.3% (8/65)	NS (0.80)
	Punctal occlusion (Stage ≥ 1)	1.5% (1/65)	4.6% (3/65)	NS (0.31)
**3 months**	Epiphora	4.6% (3/65)	7.7% (5/65)	0.088
	FDDT positive	3.1% (2/65)	10.8% (7/65)	0.048 *
	Irrigation obstruction	6.2% (4/65)	4.6% (3/65)	NS (>0.99)
	Punctal occlusion (Stage ≥ 1)	0% (0/65)	1.5% (1/65)	0.31
**6–12 months**	Epiphora	4.6% (3/65)	15.4% (10/65)	0.031 *
	FDDT positive	3.1% (2/65)	10.8% (7/65)	0.048 *
	Irrigation obstruction	—	—	—
	Punctal occlusion (Stage ≥ 1)	0% (0/65)	1.5% (1/65)	0.31

NS: not significant. *: significant (*p* < 0.05). Blank (—) indicates not measured or not applicable.

**Table 4:** Periorbital morbidity and operative metrics, Piezo vs. Classic groups (mean ± SD or *n* (%)).

Metric	Piezo (*n* = 65)	Classic (*n* = 65)	*p*-Value
Mean eyelid edema score (day 7–10)	1.5 ± 0.7	2.0 ± 0.8	0.015 *
Patients with severe edema, *n* (%) (score = 3)	3 (4.6%)	8 (12.3%)	0.04 *
Mean ecchymosis score (day 7–10)	1.3 ± 0.6	1.9 ± 0.7	0.02 *
Patients with severe ecchymosis, *n* (%)	5 (7.7%)	10 (15.4%)	0.05 *
Mean pain score (day 7–10, 0–10 NRS)	1.7 ± 1.0	2.1 ± 1.2	0.12 (NS)
Mean operative time, minutes	52 ± 10	50 ± 11	0.25 (NS)
Intraoperative complications, *n* (%)	0 (0%)	0 (0%)	—

NS: not significant. *: significant (*p* < 0.05). Blank (—) indicates not measured or not applicable.

## Data Availability

The data presented in this study are available upon request from the corresponding author. Due to privacy restrictions and institutional policy, they are not publicly archived.

## Abbreviations

LDS—Lacrimal Drainage System; FDDT—Fluorescein Dye Disappearance Test; DCR—Dacryocystorhinostomy; CT—Computed Tomography; ENT—Ear, Nose, and Throat; NRS—Numeric Rating Scale; RCT—Randomized Controlled Trial; OR—Odds Ratio; RR—Relative Risk; HR—Hazard Ratio; SD—Standard Deviation; DCG—Dacryocystography; DSc—Dacryoscintigraphy
